# Medical Opinion and Movement

**Published:** 1909-02-20

**Authors:** 


### Medical Opinion and Movement.

Lumbar puncture is a procedure that has such a wide field of use in so many affections and for so many purposes that a knowledge of the details of technique, of the dangers involved, and of the means by which they are best avoided is of the greatest importance. There has been a good deal of discussion on the question as to the amount of cerebro-spinal fluid that can be withdrawn with safety. Dr. Guinard places the limit at 20 cubic centimetres, whereas others place the figure at 30 or even 40 cubic centimetres. Dr. Le Filliatre, in discussing the matter in the *Journal de Médecine*, emphasises the importance of considering the tension of the fluid as shown by its flow from the puncture. In 300 cases of spinal anæsthesia the amount of fluid evacuated in 132 cases was less than 20 c.c., and in 168 cases the amount was between 20 and 30 c.c. If, after the puncture, the fluid flows drop by drop this author considers it sufficient to withdraw 10 to 15 c.c., according to the amount of anæsthetic he intends to inject. If, however, the fluid flows in a jet, he waits till it comes out drop by drop, and then withdraws another 10 c.c.; but in any case, even with a very high tension, he does not consider it desirable to evacuate altogether more than 30 or 35 c.c. If, in the cases of high tension, sufficient fluid is not evacuated, vomiting, headache, muscular spasms, and a general feeling of discomfort are likely to ensue. No hard and fast rule can be laid down, therefore, as to the maximum amount to be withdrawn, but the surgeon must use his discretion in each individual case.

---

Tuberculin tests have been repeatedly the subject of comment in these columns, but at the risk of being wearisome it seems worth quoting a paper in *Archives of Pædiatrics* wherein Dr. Emmett Holt describes his conclusions from 1,000 such tests in children by various methods. The statement has been made that young infants do not respond to Calmette's eye test, but this is not confirmed by Dr. Holt's experience. He thinks the skin reaction just about as reliable as the eye test, and much easier and less objectionable altogether; it has another advantage in being more characteristic and less likely to be doubtful. The puncture test and the fever reaction are probably as nearly accurate as the other two, but both are more dangerous and disagreeable. All the tests have been introduced too recently for any final decision to be arrived at. No one of them is absolutely conclusive in the sense that demonstration of the tubercle bacillus is; and each of them has given positive results in those at whose subsequent autopsy no tuberculous lesion could be found. He regards it as a great error to depend upon any of these tests to the neglect of other means of diagnosis, even though the search for the tubercle bacillus involves greater labour. Moreover, the tests do not enable us to distinguish between latent and active conditions, and, unless considered in close connection with general symptoms and physical signs, may be very misleading.

Bergmann, in a communication to the Medical Society of Riga, deals with the ætiology and treatment of spontaneous and traumatic aneurysms in the gluteal region. Aneurysms of the gluteal artery itself are not uncommon, and they are never easy lesions to diagnose. They have been mistaken, by the best surgeons, for abscesses and new growths, both of which may be difficult to differentiate from a true gluteal aneurysm, owing to the fact that the presence of pulsation in the swelling is not a certain and positive differential sign. Gluteal aneurysms are of grave prognostic significance, according to the author, as more than 50 per cent. of the cases end fatally. This is, we believe, a much higher percentage than English authorities give. The author relates a case in which the diagnosis lay between a rapidly growing sarcoma, consequent on injury, and a gluteal aneurysm in a 30-year-old patient. In this case a preliminary puncture was made on two occasions, negatively on the first, and with the emission of bright red arterial blood on the second. During the late Russian-Japanese war wounds of the gluteal artery were not rare, and many gluteal aneurysms were subsequently treated by the army surgeons. The favourite method appears to have been preliminary ligation of the iliac artery, followed by local ligature of the gluteal. Bergmann advises a much longer incision than that usually given in the books in dealing with the gluteal artery, and prefers the extra-peritoneal method for tying the iliac artery. The comparative mortality of the operation has much decreased of late years, but the difficulty in all cases is the impossibility of securing cases early enough for treatment. Commencing cases are usually diagnosed as small abscesses, and even when the tumour is large and its pulsation obvious the diagnosis is not always easy.

---

A description of the treatment of nævi and allied morbid lesions of the skin by the application of solid carbon dioxide has already been given in these columns. A further report upon the method has just appeared, by Dr. W. S. Gottheil, of New York, in the *International Journal of Surgery*. He gives some useful technical details. To obtain the solid carbon dioxide a folded towel or piece of chamois leather is firmly bandaged to the tap of the cylinder so as to leave a well-closed cylindrical space in communication with the vent. The whole must be sufficiently firm to resist a considerable pressure of gas. The first portion of gas then freezes in the interstices of the towel or leather, and the rest is then drawn under pressure through the confined space, and collects as snow or solid ice according to the degree of pressure to which it is subjected. The towel must be perfectly dry, or water ice forms in the meshes and does not hold the gas. The ice cylinder thus formed may be pointed with a knife for the purpose of application. The time of application varies from 20 to 90 seconds, or more, according as a superficial or deep cauterisation is required. The pain is said to be very slight, and the scarring, even in the cases of deeper destruction of the tissues, very satisfactory from an æsthetic point of view. The immediate effect of the application is to produce a white, hard, depressed, central area surrounded by a red elevated border. In one to three minutes the whole becomes raised and dusky red, and a little while later a tense bulla gradually forms. Left to itself the contents are absorbed, and in a few days a dry slough forms, which drops off. The method has been used successfully, especially for the destruction of nævi, but also in cases of cavernous angiomata, warts, and moles, gunpowder stains, lupus erythematosus, and even for rodent ulcer and epithelioma. For the latter, however, the results seem rather inconclusive.

Recurrence after radical cure of inguinal hernia is an event whose frequency is extremely difficult to estimate; for inquiries circulated among those who have undergone this operation produce more replies from the cured than from those in whom the hernia has recurred, many of whom betake themselves to another surgeon. Mr. W. H. Battle recently read a paper on the subject before the Medical Society of London, and he made a very telling point in referring to the favour with which the operation is now regarded by hospital patients, who throng the wards in rapidly increasing numbers anxious to have it performed. This fact shows very conclusively that the results of the modern Bassini operation are really excellent, and its extraordinary safety is demonstrated by some figures published lately by Vincenzo, who has done it 1,500 times with only one death, and that from ether pneumonia. This surgeon had but ten relapses among the 470 patients he was able to trace for a period of two years. Mr. Battle has operated for recurrence on eighteen of his own patients, though he expressly refrains from regarding that low figure as the whole truth.

While admitting that sepsis not uncommonly precedes recurrence the author is disinclined to admit that it is the chief cause. Obliteration of the sac without any pouching of the peritoneum he thinks is very important, an opinion in which one of the subsequent speakers does not concur. The strain of heavy work or violent exercise begun too soon after operation is also an important factor in favouring recurrences. Vincenzo says that general laxity and weakness of the abdominal walls is the most usual cause of relapse; but then after all this condition is very common in the subjects of hernia, whereas relapse is much less usual. Both Mr. Battle and the subsequent speakers are against the view held by Mr. Hutchinson, junr., that in operating for recurrence of inguinal hernia the testis on the affected side should be removed. One very remarkable case was quoted of a muscular man who was operated upon four times in seven years for recurrent hernias. Suppuration never happened, nor was there any obvious cause for the relapses. Every discussion upon this topic, which is so important because of the widespread incidence and disabling character of inguinal hernia, reveals steadily improving results. It is to be hoped this satisfactory progress will continue until perfection is attained.

The administration of adrenalin is recommended by Dr. W. Röhmer for the crisis of tabes dorsalis. He has tried it with success in five cases. Three of these were cases of gastric crisis, one rectal crisis, and one gastric and laryngeal accompanied by abundant salivation. The patients were given four or six drops of 1 in 1,000 adrenalin solution diluted with 20 c.c. of water three times a day. In the case of the rectal crisis the adrenalin was given per rectum after a previous evacuation with an enema. The pain and vomiting disappeared 15 to 30 minutes after the dose. The effect lasted for some time, and after several doses the crisis did not return. It seems necessary that the drug should be made to act directly upon the mucous membrane affected, and in the case of laryngeal crisis it is suggested that it should be given as an inhalation in an attenuated solution (1 in 2,000 or 5,000) by means of a nebuliser. The effect may perhaps be explained on the theory of Pál that these tabetic crises are of a vascular nature. Bottle-nose is another affection for which adrenalin is advocated by Dr. E. A. Rothmann, and also for erythematous patches of the face. The treatment must be continued for several months with short intervals. The author has found it successful in several cases, and has not encountered any undesirable symptoms either in regard to the heart or the blood pressure.

The treatment of typhoid fever by vaccines was referred to, in connection with the work of two American observers, in The Hospital on February 6, 1909, p. 476. The same idea seems to have struck Lieut.-Col. Leishman, of the Royal Army Medical Corps, at whose instance the treatment has been tried by Captain Smallman at Quetta, and the results published in the *Journal* of the Corps. Of 36 patients thus treated, three died; but two of the three had a fulminating type of disease which rapidly carried them off. It is stated that the patients had an unexpectedly good appearance, slept well, and passed large quantities of urine. The temperature, after a slight initial reaction, was soon found to commence falling, and any suspension of the fall was met by administering another dose. The paper states that everyone who has been concerned with the care of the patients feels convinced that the vaccine has a good effect; and it will be remembered that Drs. Walters and Eaton, of Boston, said very much the same thing of their own patients. Moreover, no bad effects have been discovered. Captain Smallman thinks there is little evidence to be derived from his series that the course of the disease is appreciably shortened, and here he is more cautious than the two Boston physicians who, it may be remarked, used very much smaller doses than he has done. Four of the affected men had been subjected to prophylactic inoculation, and in all four the disease was of an extremely mild type. This form of vaccine treatment must be much cheaper than Professor Chantemesse's serum treatment, from which excellent results have been reported. If it turns out on more extended investigation to bear out the experiences hitherto recorded, the treatment of enteric fever must be admitted to have made a very notable advance.

A MODIFICATION of the original Wassermann test for syphilis is published by Bauer in the *Deutsche Medizinische Wochenschrift.* The requisites of the test are, besides the serum of the suspected individual, some normal human serum; an alcohol extract of the liver of a syphilitic fœtus or infant; fresh guinea-pig serum; and a suspension in saline solution of sheep's blood corpuscles. The two human sera are first heated to 56° C., and then four small test-tubes are prepared in the following way. One contains the patient's serum with five times the quantity of liver extract and of guinea pig serum: the second has normal saline solution substituted for liver extract, and the third and fourth resemble the first two, but contain normal instead of suspected serum. After incubation at blood temperature for an hour the sheep's blood suspension is added to each. Hæmolysis occurs in the third and fourth, and more slowly in the second; whereas, if the patient is infected with syphilis, the first tube shows no hæmolysis. One of the problems connected with these serological tests for syphilis is to determine precisely at what interval after the incubation of the disease the serum reaction makes its appearance. On the analogy of the Widal test for typhoid fever some such latent period would be expected, and the point is one whose elucidation will be observed with interest. The claims that have been made on behalf of the infallibility of the Wassermann reaction have been great, but if extended research really proves that the method gives absolute assurance whether a patient does or does not harbour the virus of syphilis within him, it is difficult to name a more important recent bacteriological research. As yet, however, experts seem chary of committing themselves quite as far as this, and rightly so until more work has been done.

---

KRAMER, of Dorpat, in an article on the early diagnosis of tuberculous affections, lays great stress on the usefulness of tuberculin injections as an aid in diagnosis in these conditions. He is disposed to believe that such diagnostic injections are of as much use to the general practitioner as to the hospital specialist, especially in the case of early pulmonary tuberculosis. At the same time he insists that it is imperative that the practitioner should be intimately acquainted with the early diagnostic signs and symptoms, which are often of far greater utility in clearing up a case than even routine injections can be. In thus insisting upon the value of early signs, the author has done justice to their importance, so often overlooked in these days when the finicky "penny-in-the-slot" methods of the laboratory are apt to usurp a larger share of the practitioner's attention than they deserve. Kramer, however, expects a far greater nicety of ear than the average practitioner possesses when he demands that cases of incipient pulmonary tubercle should be positively diagnosed on the slightly increased sharpness in the inspiration sound, or the "tendency towards vesicular breathing," which, in his opinion, are the earliest signs of an apical lesion. To assert that a positive diagnosis of phthisis can be made on the strength of the auscultatory findings alone in the early stages, is to allege something which can hardly be substantiated. The specialist who has devoted years of attention and close study to mastering his stethoscope may be able to venture on a diagnosis by ear alone; even in his case, however, the verdict may be received with justifiable scepticism, unless it is backed up by concomitant evidence obtained by other means.

---

IT is this concomitant evidence that the general practitioner, who sees incipient cases at a much earlier date than does the specialist, would do well to become thoroughly familiar with. Apart altogether from the slight but important evidence furnished by careful and systematic auscultation, not merely of one apex, but of the whole thorax, with particular attention to the subclavicular and axillary regions, there are numerous small signs which, while not in themselves of much importance, are of grave significance when taken in combination with indefinite suspicions raised by the stethoscopic examination. Kramer tabulates a few of these, among them being the slight chronic laryngeal catarrh to which Schæffer has drawn attention as a very early sign in pulmonary tuberculosis; inequality of the pupils (Roques sign), owing to sympathetic irritation by swollen bronchial glands; Frederiq's sign, which consists of a slight blueish line on the patient's gums; early intermittent albuminuria; and the dilatation of superficial veins in the supraclavicular region on the affected side.

---

MOREOVER he rightly lays great stress on the importance of careful percussion; any alteration in the percussion note is to be systematically investigated. In percussion, as in auscultation, however, only constant practice can make perfect, and the practitioner would scarcely be justified in accepting the statement, emanating from recent German sources, that early cases of apical involvement are always detectable by the percussion test. Two much less generally well-known aids are given by the author. One is the asymmetry of the mammæ in women, a sign on which Richter, of Bordeaux, has lately laid stress. In early cases in female patients there is usually to be found a marked decrease in the size of the breast on the affected side; the contour of the mamma is less round, and the whole gland appears shrunken. According to Kramer this sign is of great value, as it is found in 20 per cent. of cases, but he admits that a similar asymmetry, though very rare, does occur in healthy women. Another sign on which he lays stress is also only found in female patients. It consists in a marked irregularity in the normal temperature curve, which, instead of showing the physiological pre-menstrual rise and post-menstrual fall, exhibits irregular gradations irrespective of the menstrual period in tubercular patients. This observation appears to be original, and is certainly deserving of further investigation.

